# Influence of Marital Status and Employment Status on Long-Term Adherence with Continuous Positive Airway Pressure in Sleep Apnea Patients

**DOI:** 10.1371/journal.pone.0022503

**Published:** 2011-08-17

**Authors:** Frédéric Gagnadoux, Marc Le Vaillant, François Goupil, Thierry Pigeanne, Sylvaine Chollet, Philippe Masson, Marie-Pierre Humeau, Acya Bizieux-Thaminy, Nicole Meslier, the IRSR sleep cohort group

**Affiliations:** 1 LUNAM Université, Angers, France; 2 Université d'Angers, CHU Angers, Département de Pneumologie, Angers, France; 3 CERMES, CNRS UMR8211 - Inserm U988 - EHESS, Villejuif, France; 4 Centre Hospitalier, Service de Pneumologie, Le Mans, France; 5 Pôle santé des Olonnes, Unité de Pneumologie, Olonne sur Mer, France; 6 Institut du Thorax, Pneumologie, Hôpital Laennec, Nantes, France; 7 Centre Hospitalier, Service de Pneumologie, Cholet, France; 8 Nouvelles Cliniques Nantaises, Pneumologie, Nantes, France; 9 Centre Hospitalier, Service de Pneumologie, La Roche sur Yon, France; University of Pennsylvania, United States of America

## Abstract

**Background:**

Long-term adherence is a major issue in patients receiving home continuous positive airway pressure (CPAP) therapy for obstructive sleep apnea-hypopnea syndrome (OSAHS). In a multicenter prospective cohort (the *Institut de Recherche en Santé Respiratoire des Pays de la Loire [IRSR] sleep cohort*) of consecutive OSAHS patients in whom CPAP had been prescribed for at least 90 days, we studied the impact on long-term treatment adherence of socioeconomic factors, patients and disease characteristics prior to CPAP initiation.

**Methods and Principal Findings:**

Among 1,141 patients in whom CPAP had been prescribed for an average of 504±251 days (range: 91 to 1035), 674 (59%) were adherent with a mean daily use of CPAP≥4 h (mean: 6.42±1.35 h). Stepwise regression analysis identified 4 independent factors of CPAP adherence including apnea-hypopnea index (AHI) (OR: 1.549, 95%CI 1.163 to 2.062 for AHI≥30 *vs.* AHI<30; p = 0.003), body mass index (BMI) (OR: 1.786, 95%CI 1.131 to 2.822 for BMI≥25 and <30 kg/m^2^, p = 0.01; OR: 1.768, 95%CI 1.145–2.731 for BMI≥30 kg/m^2^, p = 0.01 vs. BMI<25 kg/m^2^), employment status (OR: 1.414, 95%CI 1.097–1.821 for retired *vs.* employed; p = 0.007) and marital status (OR: 1.482, 95%CI 1.088–2.019 for married or living as a couple *vs.* living alone; p = 0.01). Age, gender, Epworth sleepiness scale, depressive syndrome, associated cardiovascular morbidities, educational attainment and occupation category did not influence CPAP adherence.

**Conclusions:**

Marital status and employment status are independent factors of CPAP adherence in addition to BMI and disease severity. Patients living alone and/or working patients are at greater risk of non-adherence, whereas adherence is higher in married and retired patients. These findings suggest that the social context of daily life should be taken into account in risk screening for CPAP non-adherence. Future interventional studies targeting at-risk patients should be designed to address social motivating factors and work-related barriers to CPAP adherence.

## Introduction

Obstructive sleep apnea-hypopnea syndrome (OSAHS) is a highly prevalent disease [1] characterized by recurrent episodes of partial or complete obstruction of the upper airways during sleep. Nasal continuous positive airway pressure (CPAP) during sleep is the primary treatment of OSAHS. Randomized placebo-controlled trials of CPAP therapy in OSAHS have demonstrated a significant benefit on daytime alertness, health-related quality of life and arterial pressure [2], [3], [4]. Observational studies have shown that CPAP therapy is associated with a lower risk of driving-related accidents [5], and fatal and nonfatal cardiovascular events [6], [7]. A number of studies have examined outcomes relative to CPAP use and have demonstrated a dose effect of CPAP therapy in improving symptoms, daytime sleepiness and quality of life [8], [9], [10]. Although there is no consistent agreement regarding the optimal CPAP use relative to health outcomes, a daily use≥4 h is frequently cited as a threshold for adequate treatment adherence [11], [12], [13], [14]. A prospective cohort study of 149 patients with OSAHS demonstrated that the greatest gain in daytime sleepiness, as assessed by the Epworth Sleepiness Scale [15], was obtained with 4 h use/night [9]. A reduced incidence of cardiovascular events under CPAP was also observed in patients using the device for at least 4 hours per night [6], [7]. Unfortunately, poor CPAP adherence is widely recognized as a critical problem in the treatment of OSAHS [16], [17], [18], [19]. When adherence is defined as greater than 4 hours of nightly use, 46 to 83% of patients with OSAHS have been reported to be non-adherent to treatment [18]. Various factors that are likely to influence CPAP adherence have been evaluated [19], including age [20], disease severity [21], [22], technical aspects [23], [24], [25], ambulatory *versus* in-hospital management [26], [27], and psychological factors [28],[29].

Recent studies have demonstrated the influence of socioeconomic status (SES) on CPAP treatment outcome [13], [30], [31], [32]. In a retrospective cohort study of 266 veterans in the USA, CPAP adherence ≥4 h/night during the first week of treatment was found to be closely associated with higher neighborhood SES [13]. In a cross-sectional study of 162 patients with newly diagnosed OSAHS in Israel, CPAP acceptance after a 2-week adaptation period was independently associated with individual SES as assessed by monthly income level [30]. Almost 30% of patients identified cost as a reason for not accepting CPAP [30] suggesting that a co-payment policy *per se* is a barrier to the purchase of CPAP in patients with low SES. Disparities in CPAP treatment outcome were also observed between OSAHS patients recruited from hospitals serving low SES neighborhoods compared with hospitals serving high SES populations [31], [32]. Forty-two percent of OSAHS patients recruited in a minority-serving institution largely treating lower income, uninsured patients failed to follow-up for CPAP treatment compared with 7% in a voluntary hospital primarily serving a middle-class population with health-care insurance [31]. In this multicenter prospective cohort study we aimed to evaluate the impact on long-term treatment adherence of socioeconomic factors, patients and disease characteristics prior to CPAP initiation.

## Methods

### Ethics statement

This study was approved by the University of Angers ethics committee and patients gave their written informed consent.

### Design and study population

Since May 15, 2007, consecutive patients ≥18 years in whom CPAP is prescribed for OSAHS in 7 centers from the west of France have been recruited in a prospective cohort (the *Institut de Recherche en Santé Respiratoire des Pays de la Loire [IRSR] sleep cohort*).

#### Inclusion criteria

All consecutive patients in whom CPAP had been prescribed for at least 90 days on April 15, 2010 were eligible for the present study.

#### Exclusion criteria

Patients with mental retardation unable to fill in the questionnaires, patients unable to give their informed consent, patients unable to read and/or speak French, and patients with neuromuscular diseases were excluded from this study.

### Baseline evaluation

Baseline evaluation prior to CPAP initiation included recording of patient characteristics, associated cardiovascular morbidities and OSAHS disease severity. Patients filled in questionnaires evaluating subjective daytime sleepiness, depressive symptoms and socioeconomic factors.

#### Patient characteristics

Patients were characterized according to their age (<65/≥65 years), gender, body mass index (BMI) (<25/≥25 and <30/≥30 kg/m^2^) and smoking habits.

#### Associated cardiovascular morbidities

Patients were classified as having cardiovascular morbidity if they reported at least one of the following cardiovascular diseases: known and treated hypertension, ischemic heart disease, cardiac arrhythmia, congestive heart failure and stroke.

#### OSAHS disease severity

Subjects were stratified by OSAHS severity based on an apnea-hypopnea index (AHI) cut-off of 30 (AHI<30/≥30 events per hour) measured by overnight polysomnography (PSG) or overnight respiratory recording. Overnight PSG was performed with continuous recording of the following channels: electroencephalogram, electrooculogram, chin electromyogram, arterial oxygen saturation (finger oximetry), nasal-oral airflow (pressure cannula), electrocardiogram, chest and abdominal wall motion (piezoelectrodes), bilateral tibialis electromyogram, and body position. Overnight respiratory recordings were performed with continuous recording of arterial oxygen saturation, nasal-oral airflow, chest and abdominal wall motion, and body position. Overnight PSG was performed under attended conditions in the laboratory, whereas respiratory recordings were performed either under attended conditions in the laboratory or under unattended conditions in hospital or at home. Respiratory events were scored manually using recommended criteria [12].

#### Subjective daytime sleepiness

Excessive daytime sleepiness was defined by an Epworth Sleepiness Scale (ESS)>10 [15].

#### Depressive symptoms

Depression was diagnosed when at least 7 items of the 13-item version of the Pichot depression scale [33] were positive.

#### Socioeconomic factors

Using specifically designed self-administered questionnaires from the Institut National de la Statistique et des Etudes Economique (INSEE), SES was described by the following variables: marital status (married or living as a couple/living alone [never married, divorced, separated, widowed]); employment status (employed full time or part time/retired/unemployed); educational attainment as determined by the age at which the patient left full-time education (≤18/>18 years); and the patient's occupational category according to the INSEE nomenclature (Farmers/Craftsman, shopkeepers, company directors/Executives and higher intellectual professions/Intermediate professions, technicians, foremen/Employees/Workers) [34].

### CPAP initiation and follow-up

The decision to prescribe CPAP was based on the following criteria: apnea-hypopnea index (AHI)≥30 events per hour or AHI between 5 and 30 events per hour with daytime sleepiness and ≥2 OSAHS symptoms including snoring, choking or gasping during sleep, unrefreshing sleep, daytime fatigue, impaired concentration, and/or nocturia. In France, CPAP treatment cost which includes delivery and follow-up by home respiratory care companies is 65% reimbursed by French national health insurance. All patients included in the present study had complementary private insurance covering the remaining 35%. Therefore, long-term CPAP therapy was provided with no additional cost to patients in the present study. A single home respiratory care company (ALISEO, Beaucouzé, France) was involved in this study for CPAP device delivery and the follow-up support program. Following the diagnosis of OSAHS, a board-certified sleep specialist prescribed CPAP therapy using either a fixed pressure device or a self-adjusting pressure device. According to French practice guidelines for OSAHS treatment [35], auto-titrating pressure devices were preferentially used in patients with sleep-stage and body position-dependent OSAHS and in those requiring high levels of CPAP. All patients were treated with devices equipped with a microprocessor and pressure monitor, providing a precise index of daily use by measuring the time spent with the mask on. For patients treated with a fixed CPAP, the effective pressure was determined either manually during titration PSG or by using the 95^th^ percentile pressure recorded during an unattended home automatic titration over at least one week [27]. Before CPAP titration, all patients received treatment education including explanation of the treatment by a specialized nurse, mask-fitting, and a CPAP acclimatization period during the daytime. All patients received a phone call from the specialized nurse during the first week of treatment and follow-up visits with the specialized nurse were then held at 3 months, 6 months and then every 6 months. According to the French recommendations for reimbursement of CPAP therapy, patients were reviewed in consultation by the sleep specialist during the first 5 months, at 12 months then at least annually. Daily CPAP use was recorded at each follow-up visit. Heated humidification was added when nasal side effects of CPAP were reported during follow-up [36]. An oro-nasal mask was used in patients with major mouth leaks under CPAP [37]. Nasal pillows were used in some patients as an alternative to nasal mask in order to provide relief to skin pressure areas, especially the nasal bridge [38].

### Primary outcome variable

The primary dependent variable of interest was CPAP adherence as assessed by mean daily CPAP use recorded at each follow-up visit. Patients were classified as CPAP-adherent when they were still using CPAP with a mean daily use of at least 4 h/night. Non-adherence corresponded to patients who refused CPAP therapy or who had stopped treatment or who were still using treatment but for an average of less than 4 h/night.

### Statistical analysis

All statistical analyses were performed with SAS software (SAS/STAT Package 2002–2003 by SAS Institute Inc., Cary, NC, USA). Adherent and non-adherent patients were compared using Chi-square test for categorical variables and 2-sample t-test for continuous variables. A logistic procedure with backward stepwise regression analysis was then used to determine independent variables influencing CPAP adherence. Only variables with a P value<0.05 were included in the model and were considered to have a significant impact on adherence with CPAP therapy. Results were expressed as mean ± standard deviation (SD) and adjusted odds ratios (95% confidence intervals).

## Results

**Figure 1:**
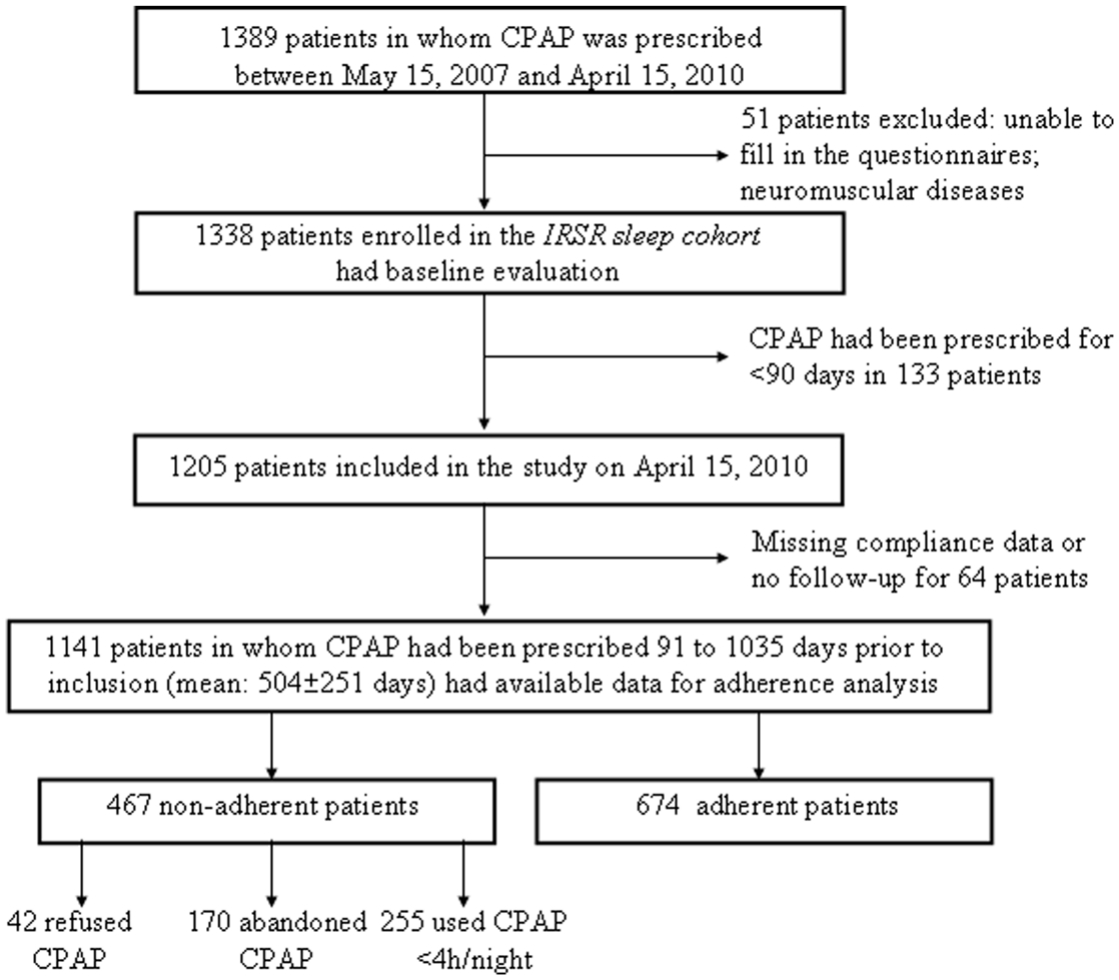
Flow diagram of subjects during the study. Abbreviations: CPAP: continuous positive airway pressure; IRSR, Institut de Recherche en Santé Respiratoire des Pays de la Loire.

A flow diagram is presented in Figure 1. Between May 15, 2007 and April 15, 2010, CPAP was prescribed in 1,389 consecutive patients with OSAHS. Fifty-one patients were excluded from the *IRSR sleep cohort* due to at least one of the abovementioned exclusion criteria. In 133 patients, CPAP had been prescribed for <90 days. Therefore, 1,205 consecutive patients, in whom CPAP had been prescribed for at least 90 days, were included in the present study. Sixty-four patients were lost to follow-up or had no available adherence data. Data from 1,141 patients in whom CPAP had been prescribed for an average of 504±251 days (range: 91 to 1035) were available for analysis. Forty-seven percent of patients were treated with fixed CPAP and 53% were treated with a self-adjusting pressure device at the time of last follow-up. A humidification system was used in 48% of cases. A total of 467 (41%) patients were considered to be non-adherent, including 42 (3.7%) patients who had refused CPAP, 170 (14.9%) patients who had abandoned treatment after a mean duration of 217±181 days and 255 (22.3%) patients who were still using CPAP, but for less than 4 h/night (mean: 2.36±1.19 h/night). Six hundred and seventy four (59%) patients were CPAP-adherent with a mean daily use of the device of 6.42±1.35 h.

Comparison of adherent and non-adherent patients (Table 1) demonstrated significant differences for BMI, AHI, marital status and employment status. Non-adherence was associated with a higher rate of employed patients, living alone, with normal weight and mild-to-moderate OSAHS, but a lower rate of obese and retired patients. There was also a trend for a higher rate of current smokers in non-adherent patients (p = 0.051). No significant difference was observed between adherent and non-adherent patients in terms of age, gender, ESS, depressive syndrome, associated cardiovascular morbidities, educational attainment and occupation.

**Table 1:** Baseline characteristics of adherent and non-adherent patients.*

Variables	Adherent patients	Non-adherent patients	P
N	674	467	
Age ≥65 years (%)	23.3	21.6	0.49
Female (%)	26	29.1	0.24
Current smokers (%)	16.6	21.4	0.05
Body mass index (kg/m^2^)	32.9 (6.7)	32.1 (6.9)	0.03
Body mass index<25 kg/m^2^ (%)	7.4	12.9	0.01
Body mass index ≥25 and ≤30 kg/m^2^ (%)	30.5	29	
Body mass index >30 kg/m^2^ (%)	62.1	58.1	
Apnea-hypopnea index	46.4 (22.1)	41.5 (21.3)	0.0002
Apnea-hypopnea index <30 (%)	19.7	29.3	0.0002
Epworth sleepiness scale	10.6 (4.9)	10.5 (5.2)	0.83
Epworth sleepiness scale <11 (%)	49.6	50.3	0.85
Pichot depression score ≥7 (%)	20.8	24.2	0.18
Patients with cardiovascular morbidity (%)	63.5	61.5	0.27
Married or living as a couple (%)	78.8	72.8	0.03
Patients who left full-time education ≤18 years (%)	71.2	69.4	0.63
Employment status			0.01
Employed full time or part time (%)	40.6	46.7	
Unemployed (%)	4.9	7.2	
Retired (%)	54.5	46.1	
Last occupation			0.75
Farmers (%)	4.1	4.1	
Craftsman, shopkeepers, company directors (%)	9.1	10.9	
Executives and higher intellectual professions (%)	15.3	15.9	
Intermediate professions, technicians, foremen (%)	14.7	16.9	
Employees (%)	14.0	14.1	
Workers (%)	26.7	23.6	
Missing data (%)	16.3	14.6	

*Results presented as mean (standard deviation) unless otherwise indicated.

Adherent patients: continuous positive airway pressure (CPAP) use ≥4 h/night.

Non-adherent patients: CPAP refused or abandoned, or CPAP use<4 h/night.

Significant level for p value: <0.05.

Multivariate analysis (Table 2) indicated that CPAP adherence was associated with 4 independent variables including AHI (OR: 1.549, 95%CI 1.163 to 2.062 for AHI≥30 *vs.* AHI<30; p = 0.003), body mass index (BMI) (OR: 1.786, 95%CI 1.131 to 2.822 for BMI≥25 and <30 kg/m^2^, p = 0.01; OR: 1.768, 95%CI 1.145–2.731 for BMI≥30 kg/m^2^, p = 0.01 vs. BMI<25 kg/m^2^), employment status (OR: 1.414, 95%CI 1.097–1.821 for retired *vs.* employed; p = 0.007) and marital status (OR: 1.482, 95%CI 1.088–2.019 for married or living as a couple *vs.* living alone; p = 0.01).

**Table 2:** Stepwise regression analysis of variables influencing CPAP adherence.

Variables	β (Standard error)	Odds ratio (95%CI)	P
AHI: ≥30 (*vs.* <30)	0.437 (0.146)	1.549 (1.163–2.062)	0.003
Body mass index (*vs.* <25 kg/m^2^)			
≥25 and <30 kg/m^2^	0.580 (0.233)	1.786 (1.131–2.822)	0.01
≥30 kg/m^2^	0.570 (0.222)	1.768 (1.145–2.731)	0.01
Employment status (*vs.* employed)			
Unemployed	−0.341 (0.284)	0.711 (0.407–1.242)	0.23
Retired	0.346 (0.129)	1.414 (1.097–1.821)	0.007
Marital status: Married or living as a couple (*vs.* living alone)	0.393 (0.158)	1.482 (1.088–2.019)	0.01

Abbreviations: CPAP, continuous positive airway pressure; AHI, apnea-hypopnea index;

CPAP adherence: CPAP use ≥4 h/night.

Significant level for p value: <0.05.

Area under the ROC: 0.607.

## Discussion

In this multicenter prospective cohort study, 59% of 1,141 OSAHS patients where CPAP-adherent with a mean daily CPAP use ≥4 h an average of 504 days after the initial prescription. Our findings support an independent influence of marital status and employment status on long-term CPAP adherence. In line with previous reports [20], [21], we also demonstrated that BMI and OSAHS severity are independent predictors of long-term CPAP adherence.

Few studies have evaluated the influence of social support (mainly by partner) on CPAP adherence. In a prospective cohort study of 80 consecutive OSAHS patients, Lewis et al. [39] found that those subjects who lived alone used their machines significantly less than those who lived with a partner, suggesting that living with another person may encourage regular CPAP use. Simon-Tuval et al. [30] demonstrated that social support from family and/or friends' positive experience with CPAP was an independent predictor to increase the odds of CPAP acceptance (OR = 2.6 and 2.9 for the whole group and patients living with a partner, respectively). The bed partner's post-treatment sleep quality and overall quality of life were also demonstrated to influence CPAP adherence [40]. In a small population of married men, CPAP adherence was strongly related to the frequency with which the couple slept together [41]. Recent studies have more extensively investigated how the social context of daily life may impact on perceptions of CPAP treatment [42], [43]. Married OSAHS patients described close sources of support (i.e., spouse, living partner, family members) as important to provide feedback about their response to treatment, troubleshooting difficulties and positive reinforcement for persistent CPAP use [43]. A study of spousal involvement in CPAP adherence among 31 OSAHS patients found that the patient's perception of the wife's support predicted increased adherence in patients with high disease severity [42]. Increased positive wife involvement occurred as a reaction to adherence and problems with CPAP [42]. Our findings and those of previous research [30], [39], [42], [43] suggest that marital status should be taken into account in risk screening for CPAP non-adherence. Further studies are needed to evaluate supportive living partner involvement as an adherence intervention. In unmarried OSAHS patients, family members, friends and/or coworkers could constitute social support resources and be involved in future educational strategies to improve CPAP adherence.

The independent effect of employment status on treatment adherence has been demonstrated in various disease settings. A prospective cohort study of patients undergoing warfarin therapy showed an increased risk of non-adherence in patients currently employed compared to unemployed and retired patients [44]. Among patients with inflammatory bowel disease, men with lower medication adherence were more likely to be employed on a full-time basis [45]. Work-related barriers including being away from home and being too busy or distracted to properly comply were also identified in patients with HIV infection receiving highly active antiretroviral therapy [46]. Work performances have been demonstrated to be impaired in OSAHS patients with excessive daytime sleepiness and to improve in response to CPAP treatment [47]. Unfortunately, the present study demonstrates that employed OSAHS patients are at greater risk of CPAP non-adherence compared to retired patients. Although the underlying relationship is not certain, active employment might reflect numerous competing interests which take precedence over regular CPAP use. CPAP machines are often considered to be bulky, which can contribute to limit CPAP adherence in patients travelling for work. Furthermore, conflicting demands imposed by work schedules may compromise long-term CPAP follow-up visit attendance. Further studies should be designed to better address work-related barriers to CPAP adherence.

Two recent studies found that economic status, as assessed by income level [30] and neighborhood of residence [13], is an independent factor of CPAP adherence. Treatment cost was identified as a reason for declining CPAP by 30% of patients [30] suggesting that co-payment policy may contribute to the negative impact of low SES on CPAP acceptance. As income levels and neighborhood of residence were not measured in the present study, the potential influence of these parameters on CPAP adherence cannot be excluded. However, no link was observed between long-term CPAP adherence and two of the variables defining economic status, i.e. educational attainment and occupational category. In the present study, CPAP therapy was provided with no additional cost to patients. It can therefore be hypothesized that the absence of a co-payment policy may have attenuated the influence of economic factors on CPAP adherence.

In line with previous reports, we found no independent influence of age [20], [21], [22] and gender [21], [22] on long-term CPAP adherence. We and other authors have found that obesity is an independent predictive factor of better CPAP adherence [20]. In the large study from McArdle et al. [21], obesity was not an independent predictive factor of CPAP adherence, but increasing BMI was a significant determinant of the number of hours of use of the device per night. The underlying relationship between BMI and CPAP adherence is unclear. Previous investigations of health belief model in OSAHS [29] found that higher BMI prior to CPAP treatment was associated with greater functional limitations including lower activity levels, poorer vigilance and lower productivity throughout the day. It can be hypothesized that higher perceived functional limitations due to OSAHS in overweight and obese patients contribute to increase CPAP adherence, but this remains to be demonstrated.

Our results corroborate the findings of most previous investigations demonstrating that the severity of sleep-disordered breathing, as assessed by AHI or oxygen desaturation index, is a determinant of long-term CPAP use [20], [21], [22], [30]. Conversely, daytime sleepiness prior to CPAP treatment, as assessed by ESS, is an inconsistent predictor of adherence in the literature [29]. In contrast to the study from McArdle et al. [21], we did not find that non-sleepy patients with ESS<11 at diagnosis are less likely to adhere to CPAP, although the two studies are comparable in terms of sample size and the rate of non-sleepy patients (40–50%). Furthermore, no link was demonstrated between depressive symptoms and CPAP adherence in our study. Several recent studies have also failed to demonstrate any influence of ESS and psychological variables on CPAP adherence [13], [22], [29], [30] suggesting that the severity of daytime sleepiness does not play a pivotal role in terms of long-term treatment adherence. Post-treatment perception of an improvement in ESS was found to be predictive of ongoing CPAP use, but is of limited value for the identification of patients likely to present poor adherence prior to initiation of therapy [19].

This study presents a number of limitations. The impact of technical factors and initial CPAP exposure factors on treatment adherence was not evaluated [19]. Technical aspects such as CPAP mode, humidification or interface were likely to be modified during treatment follow-up. These parameters are therefore of limited value in the prediction of treatment adherence prior to CPAP initiation. Regarding initial CPAP exposure factors, recent prospective randomized studies failed to demonstrate any impact of ambulatory versus in-hospital management on CPAP treatment outcome [26], [27]. Despite the prospective design of this study, the missing data rate was about 15% for occupational categories that might have contributed to a selection bias. However, the missing data rate was similar in adherent and non-adherent patients.

In conclusion, marital status and employment status are independent factors of CPAP adherence in addition to BMI and disease severity. Patients living alone and/or working patients are at greater risk of non-adherence, whereas adherence is higher in married and retired patients. These findings suggest that the social context of daily life should be taken into account in risk screening for CPAP non-adherence. Future interventional studies targeting at-risk patients should be designed to address social motivating factors and work-related barriers to CPAP adherence.
